# Laryngeal tuberculosis diagnosed by stool sample cultures: a case report

**DOI:** 10.1186/s13256-015-0548-1

**Published:** 2015-03-31

**Authors:** Nicolas Yin, Marion Delord, Antoine Giovanni, Jean del Grande, Michel Drancourt, Philippe Brouqui, Jean-Christophe Lagier

**Affiliations:** 1 Assistance Publique-Hôpitaux de Marseille, CHU Nord, Pôle Infectieux, Institut Hospitalo-Universitaire Méditerranée Infection, 13015 Marseille, France; 2 Service d’ORL et de chirurgie cervico-faciale, CHRU La Timone, 264, rue Saint-Pierre, 13385 Marseille, cedex 05 France; 3 Service d’Anatomo-pathologie, Assistance Publique-Hôpitaux de Marseille, Hôpital de la Timone, Marseille, France; 4 Aix Marseille Université, URMITE, UMR63, CNRS 7278, IRD 198, Inserm 1095, Marseille, France

**Keywords:** Diagnostic test, Dysphonia, Laryngeal tuberculosis, Stool culture

## Abstract

**Introduction:**

Laryngeal tuberculosis is a rare and often misdiagnosed disease. Its diagnosis is based on the association of a laryngeal lesion and the microbiological detection of *Mycobacterium tuberculosis*. Stool cultures have recently been described as a useful tool in the diagnosis of atypical forms of tuberculosis. In this report, we describe the first case in the literature of laryngeal tuberculosis diagnosed by culture of stool samples.

**Case presentation:**

A 41-year-old French Caucasian man was admitted to our hospital for dysphonia of 3 months’ evolution. A laryngeal biopsy was performed because of suspicion of carcinoma. He had no clinical signs of tuberculosis. The biopsy showed a caseating granuloma suggestive of laryngeal tuberculosis. The diagnosis was finally confirmed by stool cultures, whereas sputum cultures remained sterile for *M. tuberculosis*.

**Conclusions:**

This case confirms the importance of stool cultures in the diagnosis of tuberculosis, especially for patients with uncommon presentations.

## Introduction

Laryngeal tuberculosis is a rare form of clinical presentation of tuberculosis in Western Europe, where it accounts for less than 1% of cases [1,2]. Frequently, symptoms are limited to dysphonia or odynophagia, and the usual symptoms of tuberculosis, such as fever, cough and night sweats, are absent [1,2]. In many published cases, a diagnosis of carcinoma was suspected first and then rejected after the observation of caseating granuloma in histological analysis [3]. The microbiological confirmation is obtained by culture and smear microscopy of sputum. In recent years, analytical tools for use with stool samples with smear microscopy, culture and polymerase chain reaction (PCR) have been developed, effectively replacing gastric aspiration in patients with pulmonary tuberculosis [4,5]. In this report, we present a case of a 41-year-old man with laryngeal tuberculosis whose microbiological diagnosis was obtained by stool culture while sputum and bronchial washing cultures remained negative.

## Case presentation

**Figure 1:**
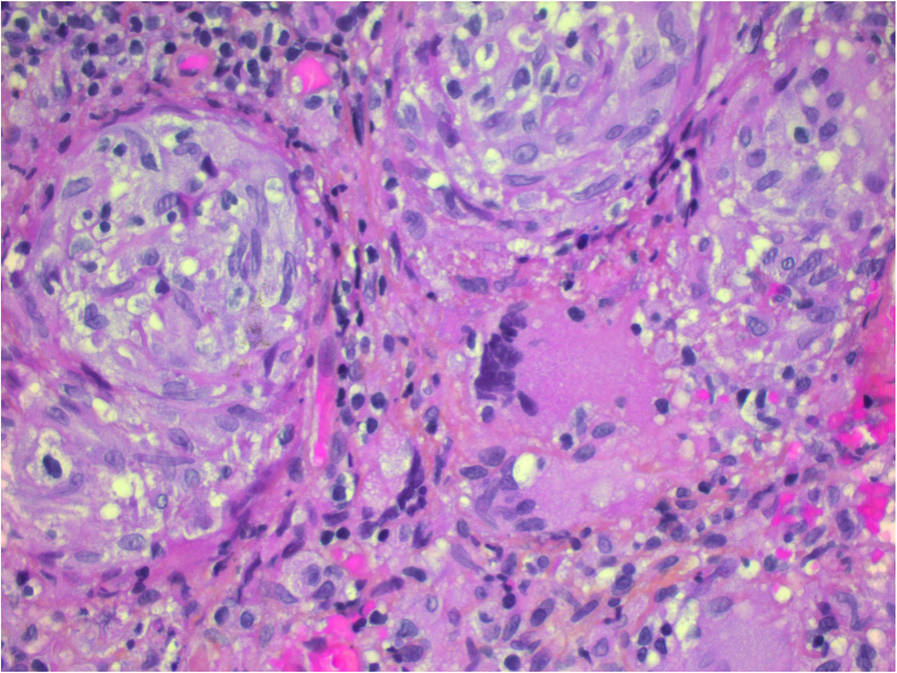
**Hematoxylin phloxine saffron stain of the patient’s: epithelioid histiocytic granuloma and multinucleated giant cells (original magnification X 400).**

A 41-year-old French Caucasian man consulted an ear, nose and throat specialist for dysphonia of 3 months’ evolution that was associated with persistent asthenia. He had been given the Bacillus Calmette–Guérin vaccine and had had a limit tuberculin skin test of 10mm 20 years before presentation. No immunosuppressive treatment had ever been prescribed, and his serological status for HIV and hepatitis was negative. The patient reported no fever, night sweats or significant weight loss. His clinical examination was normal, excepting dysphonia without cough or dyspnea. His dysphonia was characterized by hoarseness and a loss of voice concomitant with increasing asthenia. A laryngoscopy examination revealed an ulcerated lesion of the posterior part of the left vocal cord with a suspected malignant macroscopic aspect. Histological analysis of a laryngeal biopsy showed intense acute inflammatory lesions associated with tuberculoid granuloma without carcinoma (Figure 1). The patient was then admitted to the hospital.

**Figure 2:**
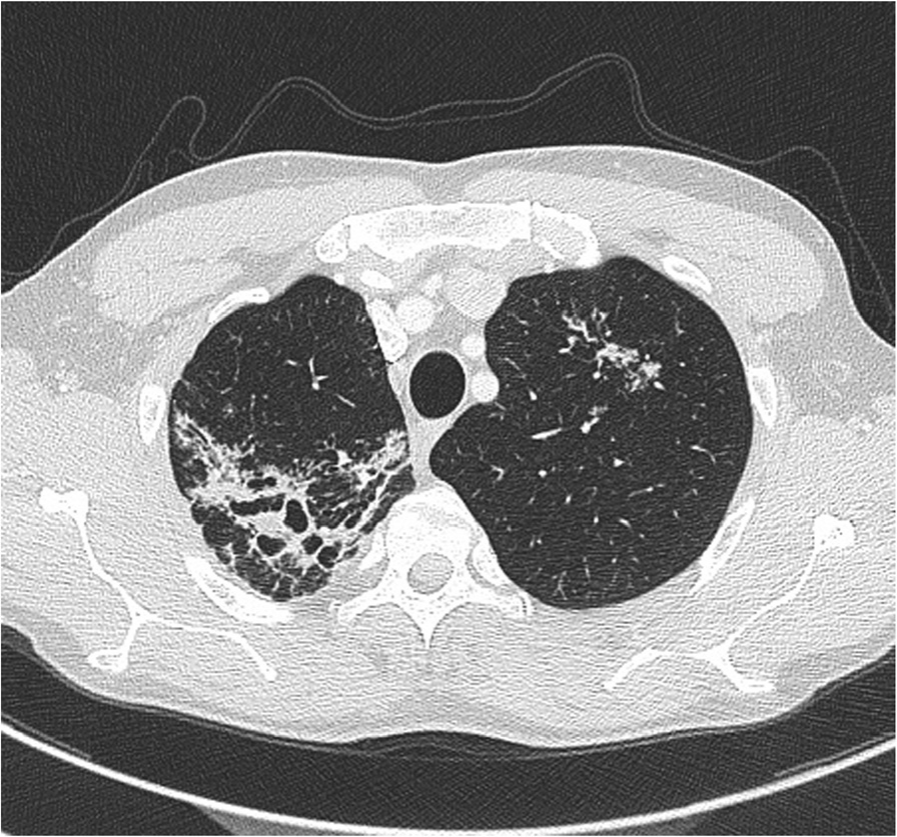
**Thoracic computed tomographic scan showing a lesion suggestive of tuberculosis in the right apex and diffuse micronodular involvement.**

The results of his routine biological examination were normal. Chest computed tomography (CT) revealed micronodular lesions in both lungs and a lesion suggestive of tuberculosis in the right apex (Figure 2). Sputum, urine and stool samples were collected every day for 3 consecutive days for mycobacterial smear microscopy and culture. Bronchial washing was performed. All direct microscopic examinations remained negative. Stool cultures collected at day 2 and day 3 grew after 29 days of culture on MGIT™ (BD Diagnostic Systems, Le Pont de Claix, France), although sputum and bronchoalveolar lavage cultures remained negative at 45 days. PCR performed on colonies identified *Mycobacterium tuberculosis*. Antibiotic testing showed susceptibility to rifampicin, isoniazid, ethambutol, pyrazinamide and streptomycin. Consequently, the patient was treated with an antibiotic regimen associating rifampicin (10mg/kg/day), isoniazid (5mg/kg/day), pyrazinamide (30mg/kg/day) and ethambutol (20mg/kg/day) for 2 months, then rifampicin (10mg/kg/day) and isoniazid (5mg/kg/day) for 4 months. After 5 months of well-tolerated treatment, the patient totally recovered.

## Discussion

In our patient, as in many cases of laryngeal tuberculosis previously described [1-3], the diagnosis was delayed because of the unusual clinical presentation. No symptom of pulmonary tuberculosis was present, and dysphonia was isolated. In a review, Benwill *et al*. showed that dysphonia is the main common symptom, followed by weight loss [1]. Tobacco smoking and alcoholism are frequent comorbidities [1]. This clinical presentation is frequently confounding, especially in developed countries, where the first suspected diagnosis is carcinoma in cases with such clinical involvement [3]. Moreover, the endoscopic aspect is not specific in many cases. In our patient, histological analysis guided the diagnosis, but as tuberculosis had not been suspected, neither surgical biopsy nor peripheral samples were analyzed in the microbiology laboratory before the histological results were obtained. The biopsy was fixed with the procedure used for carcinoma, which decreased the chance of identifying mycobacteria. Retrospective detection of *M. tuberculosis* on vocal cord biopsy by PCR and Ziehl–Neelsen staining remained unsuccessful.

A pure culture remains essential to test antibiotic susceptibility, especially because of the appearance of multidrug-resistant tuberculosis [6]. Recent progress in culture media and culture conditions have successfully accelerated mycobacterial diagnosis [7,8]. The optimization of blood agar media using different compounds, such as egg lecithin and the addition of ascorbic acid, has a growth-promoting effect [7]. The use of microaerophilic conditions and the detection of microcolonies using autofluorescence succeeded in dramatically decreasing the culture time of *M. tuberculosis* [7].

In parallel, to improve the diagnosis of tuberculosis, we used systematically a diagnostic kit developed in our laboratory [9] with which we simultaneously collected sputum and stool samples during a 3-day period. Indeed, in this case, although chest CT suggested lung tuberculosis, *M. tuberculosis* was not detected in respiratory tract samples, including samples obtained by bronchial washing. The microbiological diagnosis was only performed because of the positive culture on two stool samples with identification confirmed by PCR. To the best of our knowledge, this case constitutes the first case in the literature of laryngeal tuberculosis diagnosed microbiologically by stool sample analysis. This underlines the necessity of using a comprehensive approach to the diagnosis of tuberculosis facilitated by the use of this kit [9]. Stool sample analysis is a non-invasive procedure useful for helping to diagnose tuberculosis [4,5]. Some studies have shown that it can be a rapid tool, especially in children with HIV infection, with the use of PCR [10,11]. Finally, a large study suggested that stool culture was similar to third sputum for diagnosing tuberculosis in patients with HIV infection [12].

## Conclusions

This case confirms the usefulness of routine stool culture in making the diagnosis of tuberculosis, including atypical presentations.

## Consent

Written informed consent was obtained from the patient for publication of this case report and any accompanying images. A copy of the written consent is available for review by the Editor-in-Chief of this journal.

## Abbreviations

CT: Computed tomography
PCR: Polymerase chain reaction
